# Accurate, Model-Based Tuning of Synthetic Gene Expression Using Introns in *S. cerevisiae*

**DOI:** 10.1371/journal.pgen.1004407

**Published:** 2014-06-26

**Authors:** Ido Yofe, Zohar Zafrir, Rachel Blau, Maya Schuldiner, Tamir Tuller, Ehud Shapiro, Tuval Ben-Yehezkel

**Affiliations:** 1 Department of Molecular Genetics, Weizmann Institute of Science, Rehovot, Israel; 2 Department of Biomedical Engineering, Tel-Aviv University, Tel Aviv, Israel; 3 Department of Applied Mathematics and Computer Science, and Department of Biological Chemistry, Weizmann Institute of Science, Rehovot, Israel; 4 The Sagol School of Neuroscience, Tel-Aviv University, Tel-Aviv, Israel; The University of North Carolina at Chapel Hill, United States of America

## Abstract

Introns are key regulators of eukaryotic gene expression and present a potentially powerful tool for the design of synthetic eukaryotic gene expression systems. However, intronic control over gene expression is governed by a multitude of complex, incompletely understood, regulatory mechanisms. Despite this lack of detailed mechanistic understanding, here we show how a relatively simple model enables accurate and predictable tuning of synthetic gene expression system in yeast using several predictive intron features such as transcript folding and sequence motifs. Using only natural *Saccharomyces cerevisiae* introns as regulators, we demonstrate fine and accurate control over gene expression spanning a 100 fold expression range. These results broaden the engineering toolbox of synthetic gene expression systems and provide a framework in which precise and robust tuning of gene expression is accomplished.

## Author Summary

Synthetic biology is gradually expanding our capability to engineer biology through rational genetic engineering of synthetic gene expression systems. These developments are already paving the way for the accelerated study of biology and applying engineered biological systems to major environmental and health problems. However, our capacity to intelligently modify and control gene expression depends on our ability to apply a broad range of genetic regulators in the engineering process. Here we show that Introns, pivotal regulators of Eukaryotic gene expression, can be rationally engineered to control a synthetic gene expression system of a Eukaryote. We developed a unique reporter-based system to evaluate the effects of engineering splicing in synthetic biology and show that the entire intron repertoire of *S. cerevisiae* can be accurately used to rationally engineer gene expression. Our results provide both a proof-of-concept for the integration of splicing into synthetic biology designs and a model that can be used by the scientific community for integrating splicing into their own designs. Following the extensive use of transcriptional (promoter) and translational (UTR) elements in synthetic constructs, our results introduce a new major regulatory system, splicing, that can be used to rationally engineer genetic systems.

## Introduction

Advancements and innovations in synthetic and computational biology have revolutionized our ability to rationally engineer libraries of single synthetic genetic elements (such as promoters or ribosome binding sites) and have increased our capacity to finely tune the expression of genes according to specification. Additionally, the rational tailoring of synthetic gene networks is gradually enabling the engineering of more complex genetic behaviors and control over various features of gene expression by altering a cells genetic code [1]–[5] or its extracellular signal concentrations [6]. Nevertheless, establishing reliable rules for applying regulatory genetic elements in the engineering of synthetic gene expression systems is still a major challenge in synthetic biology. One obstacle to reaching this goal is a lack of well-characterized genetic parts that can be readily used to accurately and predictably control gene expression in synthetic genetic contexts [7]–[10]. Gene expression is affected by a myriad of trans acting factors as well as interdependent cis regulatory elements such as promoters, upstream and downstream untranslated regions (UTR's) and introns. Since splicing of introns must be performed before translation can begin, it is a key step in controlling gene expression. However, deciphering how splicing regulation is encoded within pre-mRNA transcripts has proven to be a major challenge [11], [12]. As a result, introns have been largely absent as a genetic “part” that can be integrated into the design of synthetic cellular systems. In this study we broaden the repertoire of genetic elements for bio-engineering by showing how introns can be used to regulate gene expression in a synthetic gene.

**Figure 1:**
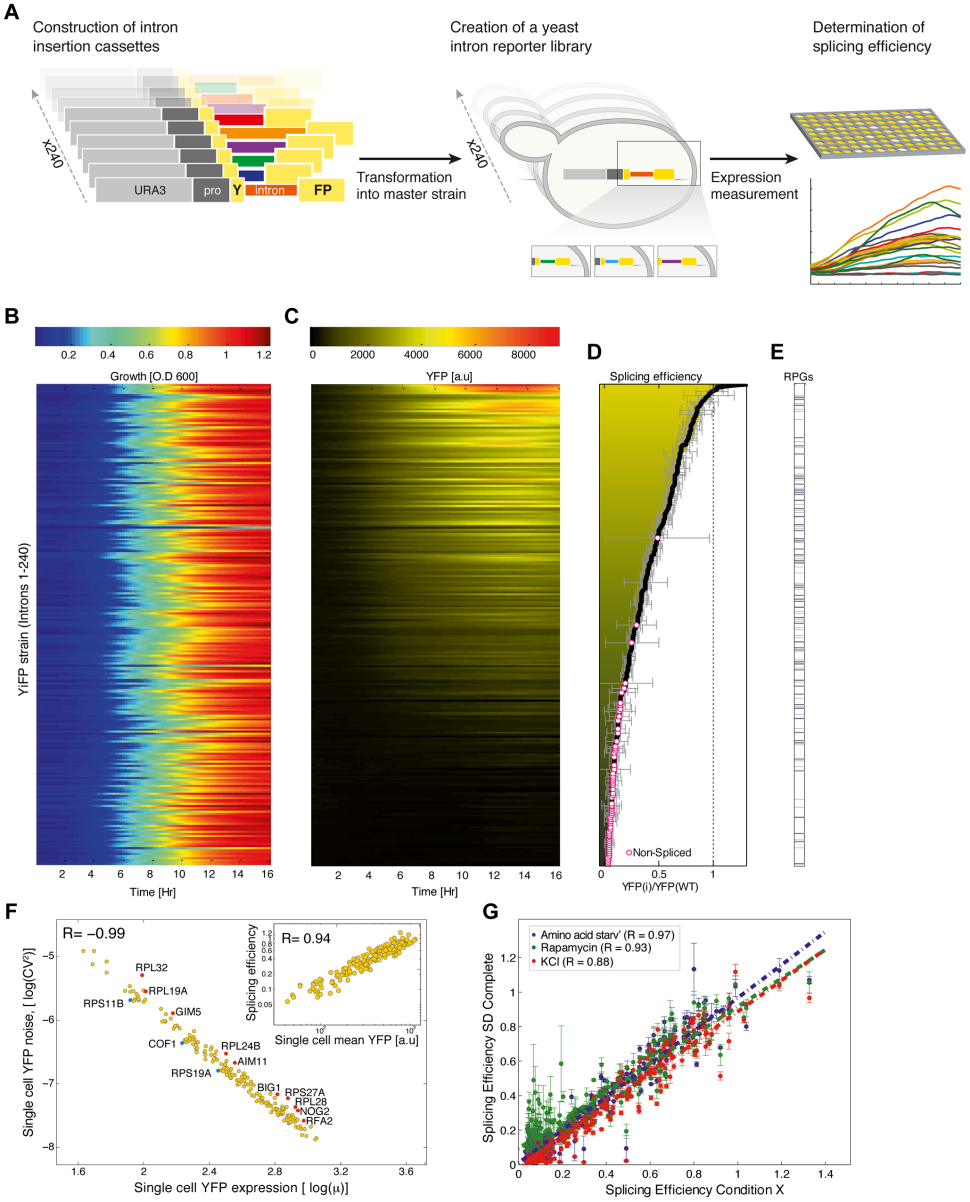
A standardized reporter library uncovers splicing regulatory information encoded within introns. A) Overview of the reporter approach for studying splicing mediated gene expression regulation. Intron insertion cassettes were constructed *in-vitro*, each comprised of a selection marker (URA3), a constitutive promoter, the first 195 nucleotides (nt) of the YFP gene, and one of 240 native *S. cerevisiae* introns followed by an additional 60 nt of the YFP gene. Each insertion cassette was transformed into the genome of a master strain which contained a promoter-less YFP gene, thus creating an *in-vivo* intron-reporter yeast library (YiFP). Culture growth and YFP expression levels of each variant in the library were monitored using a micro-plate reader. B) All strains in the YiFP reporter library grew similarly. C) However, each intron conferred unique YFP expression levels. D) Each strain's average expression levels, YiFP(i), were compared to that of an intron-less reference strain, YFP(wt), to get an assessment of “splicing efficiency”. YiFP strains whose YFP levels did not pass the detection limit were considered as “non-spliced” (marked with circles). Error bars represent ±1 SD from four independent experiments. E) Splicing efficiency of ribosomal and non-ribosomal protein genes (RPGs) is distributed in a similar manner. F) Analysis of YFP expression at the single cell level (using automated microscopic imaging) validated splicing efficiency measurements of spliced introns (inset graph, r = 0.94), and enabled assessment of splicing efficiency noise in a population. Noise is represented by the (squared) YFP expression coefficient of variation (CV^2^), *i.e.* the variance (σ^2^) normalized by the squared mean YFP expression (μ^2^), for each intron strain as determined using microscopic imaging analysis. Gene names of introns that presented noise higher (red) or lower (blue) than normal are indicated (outliers of linear regression; p<0.1). G) Splicing efficiency in a synthetic context is robust to environmental change. Yeast were grown in several stress conditions (Amino acid starvation, Rapamycin, 1M KCl) known to affect the splicing machinery. Error bars represent ±1 SD from three independent experiments.

We have constructed a synthetic gene expression library that tests the effect of most of *S. cerevisiae*'s native introns with a quantitative fluorescent output (Figure 1A), enabling *in vivo*, dynamic monitoring of intron-mediated regulation of gene expression in a synthetic gene context. Surprisingly, despite the mechanistic complexity of intronic splicing and of the splicing code, analysis of expression data from this novel library shows that a simple statistical model that integrates the few major known regulatory determinants of intron splicing in and around introns (such as RNA secondary structure, GC content and sequence motifs) accounts for the vast majority of gene expression variability observed when integrating many different introns into a synthetic gene expression system. The predictability of intron's effects is a major advantage in utilizing such elements for engineering purposes.

## Results

### A synthetic reporter library uncovers rules for accurately engineering intron splicing into synthetic gene expression systems

To create a synthetic intron reporter library we transformed yeast with a library of DNA transformation cassettes each containing a different native yeast intron. The cassettes were assembled using the Y-operation [13], [14] by which introns were embedded in a Yellow Fluorescent Protein (YFP) fragment and concatenated to a common selection marker in high throughput (Figure 1A, Materials and methods, and figure S1). In this manner 240 strains were created, termed YiFP strains, where the sole difference between all strains is the native *S. cerevisiae* intron intervening the YFP gene. Introns were positioned in the YFP so as to both eliminate false positive splicing signals (Text S1) and to mimic the natural location of introns in their endogenous context, which in *S. cerevisiae* is biased towards the 5′ end of the coding sequence [5].

We then assessed the contribution of introns to the regulation of gene expression by dynamic measurements of YFP expression. The entire library was cultured in 384-well plates together with reference strains harboring an intron-less YFP (YFP-wt), and strains that had no YFP altogether. We monitored culture growth (O.D_600_) and YFP fluorescence of each strain for 24 hours using a micro-plate reader, in four independent replicates. Our analysis shows that while growth characteristics remained coinciding for almost all intron library strains and controls (Figure 1B, Text S1, table S2 and figure S9 and Table S8 for outliers), YFP expression spanned over two orders of magnitude (Figure 1C, figures S2 & S3 and figure S10 and Table S9 for outliers). Strains that had a signal-to-noise ratio (SNR) below 5 were classified as un-spliced. Importantly, YFP fluorescence levels were validated to consistently reflect YFP mRNA levels using quantitative real time PCR (qPCR) (figure S4; r = 0.99; p = 2.3e-04). Following normalization, the expression level of each intron strain was compared to that of the intron-less YFP strain to give a measure of relative expression level, that we relate to as “splicing efficiency” (Figure 1D and table S1).

Interestingly, YiFP strains expression data shows that introns almost exclusively reduce reporter gene expression compared to the intron-less YFP reference strain (Figure 1D, Splicing efficiency <1). This finding highlight the differences between yeast and mammalian cells in which introns boost gene expression [15], [16]. In addition, we observed that simple intron features such as intron length could not account for the variability in gene expression recorded in our library (table S6). For example, *S. cerevisiae* ribosomal protein genes (RPGs) introns are substantially longer than introns of non-RPG's, with means of 400 and 100 base pairs (bp) respectively; however, RPGs introns were not clustered to higher or lower splicing efficiencies in our library (Figure 1E). Conversely, intron features known to significantly affect intron function such as secondary structure and GC content at intron-exon junctions, as well as certain sequence motifs were found to be dominant intron features that dictate splicing efficiently in a completely synthetic system.

### Single cell expression analysis validates splicing efficiency measurements, and allows the assessment of population-level variability

To assess how much of the changes in splicing efficiency stem from a wide distribution of splicing capacity in the population vs how much stems from single cell behavior, we performed high-throughput single cell analysis for all 240 library strains using automated microscopy imaging. We explored whether splicing efficiency of single cells from intron strains correlate with our splicing efficiency index (Materials and methods and figure S5). Results show that average expression from single cells is highly correlated with our splicing efficiency index (Figure 1F, inset; r = 0.94) and that noise in YFP expression, i.e. cell-to-cell variability, within strains is highly correlated with the expression levels of single cells from the same strain, as also observed for fluorescently tagged yeast proteins [17]. This characteristic may play a role in setting a lower bound to the degree one can reliably down-regulate gene expression with introns. Nevertheless, we did identify a few introns that confer a lower or higher noise level than expected (Figure 1F, marked in blue and red, respectively).

### Introns in a synthetic gene context are resistant to changes in environmental conditions

The splicing of specific subsets of pre-mRNAs is modulated in response to various environmental conditions [18], [19]. Interestingly, our results show that introns embedded within a synthetic gene expression system and exposed to four different conditions known to elicit changes in splicing levels do not respond accordingly, despite the fact that the change in growth condition was indeed being registered by the cells [18], [19] (Figure 1G and tables S2 & S3). The loss of condition-specific splicing in synthetic expression systems indicates that introns are not sufficient for encoding splicing specificity. Additionally, in contrast to classes of genetic elements (such as promoters) that contain variants that are environmentally responsive, it seems that the entire repertoire of *S. cerevisiae* introns is insulated from environmental changes and may be used as robust regulators in changing environments.

### Intronic sequence motifs tune synthetic gene expression systems

**Figure 2:**
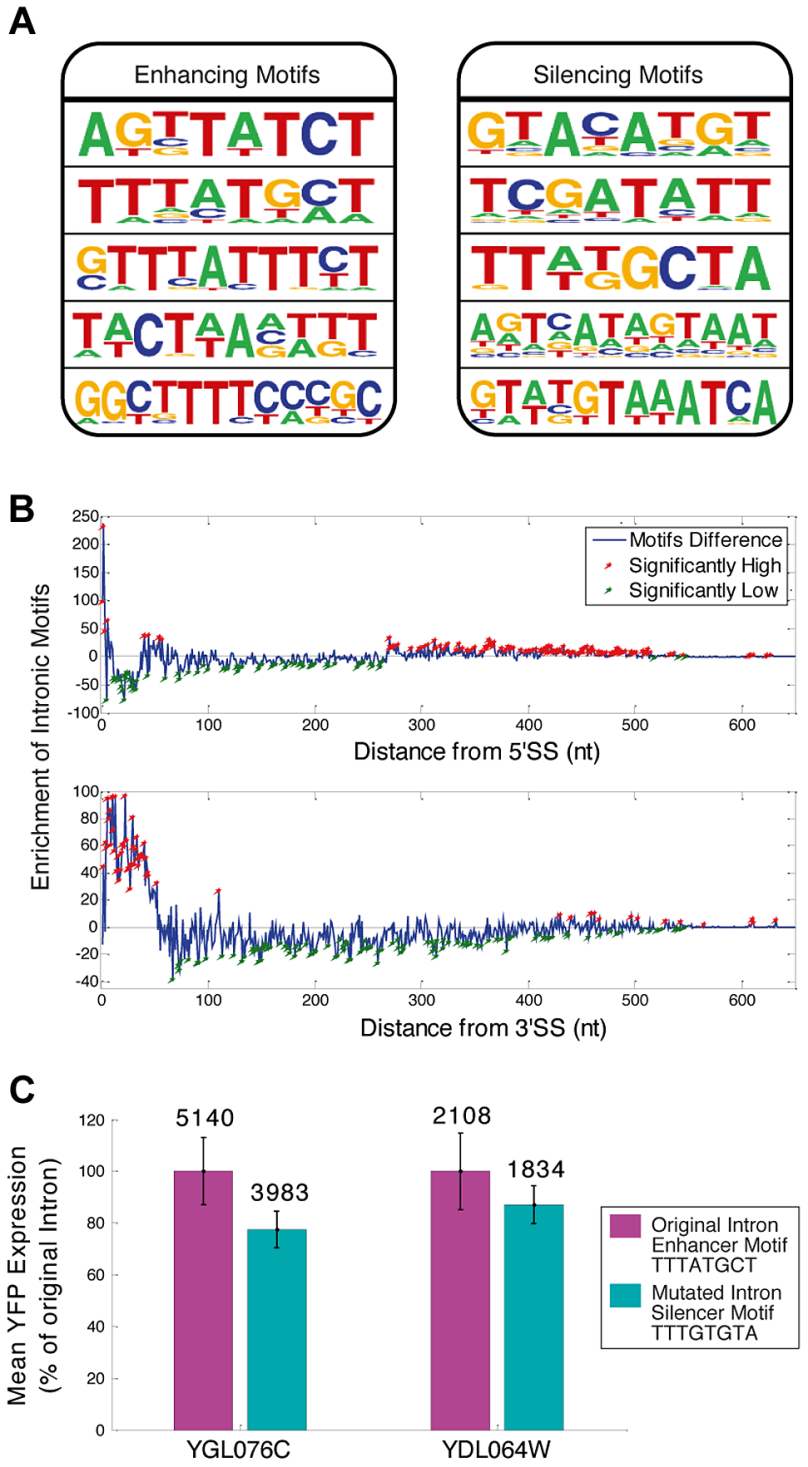
Sequence motifs function “out of context”. A) Motifs associated with splicing efficiency were revealed by comparing intron sequences of high and low expressing YiFP strains. Examples of five enhancers (enrichment p<3.4e-4) and silencers (p<4e-4) are shown. B) The novel motifs were enriched in proximity to intron ends. Enrichment of motifs in introns compared to a randomized/permutated version of the motifs that maintain their properties (blue line, Materials and methods) is presented in respect to distance from 5′- or 3′ Splice Site (SS) (top and bottom, respectively). Positions significantly enriched or deprived of motifs are marked in red and green, respectively. C) Reporter YFP expression is decreased upon exchange of an enhancer to a silencer motif in two independent intron strains. Mean YFP expression was calculated for triplicates of the two intron library strains (YGL076C- p = 0.02, and YDL064W- p = 0.2). Average expression of each mutated motif strain is shown in comparison to that of the natural intron harboring YiFP strain (100%). Numbers inset in bars indicate the mean YFP expression level (non-relative).

In addition to the canonical splicing signals (5′ & 3′ splice sites (SS) and branch point (BP)), which participate in splicing chemistry, splicing regulatory elements (SREs) within exons and introns are key factors that determine splicing efficiency and expression levels in higher Eukaryotes [2]–[5], [20]. Evidence for SRE function in *S. cerevisiae* has been gradually emerging in recent years [3]. We used our library expression data to identify SREs and found ISEs and ISS motifs (Figure 2A). We analyzed the positional distribution of motifs along the 240 introns of the library and found that the motifs are highly enriched near both splice sites (Figure 2B, figure S6 and Table S4). In order to test whether indeed the motifs can be used as independent entities to regulate intron dynamics we performed directed mutagenesis to the enhancer motif TTTATGCT in three nucleotides, transforming it into the silencer motif TTTGTGTA in two independent introns in two YiFP strains. Transforming these enhancers to silencers resulted in a reduction of 22% and 13% in their expression levels compared to the enhancer containing introns (Figure 2C). This proof of principle opens possibilities for large scale re-encoding of introns with sequence motifs, demonstrating the mobility and utility of splicing motifs that reside within introns for engineering gene expression in synthetic systems.

### RNA secondary structure at artificial intron-exon junctions dictates gene expression

**Figure 3:**
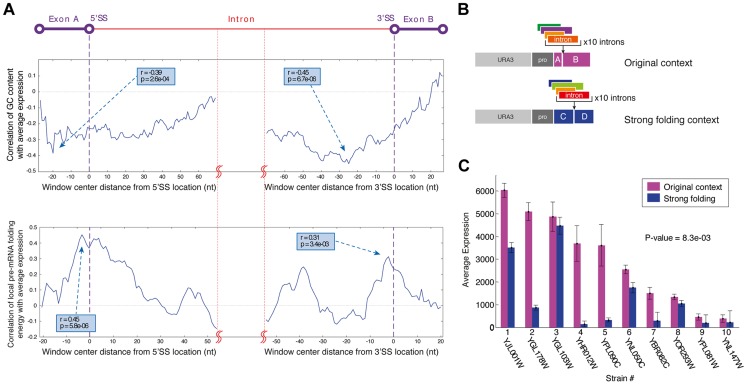
The exonic context of introns is a major regulatory determinant of gene expression. A) Profiles of the correlation of GC content (top) and local mRNA folding energy (bottom) around the 5′SS and 3′SS with YiFP expression levels identified these features as determinants affecting gene expression. Sliding window sizes are 50 nt for GC content and 40 nt for folding energy. B) Ten introns were inserted into a stronger folding location in the YFP to test this feature's effect on gene expression. C) Introns inserted into a location within the YFP reporter with stronger folding (blue) confer lower expression levels. Averages of three independent experiments are presented (paired *t* test p = 8.3e-03).

The cross-talk between introns and their surrounding exonic sequences regulates splicing through the formation of RNA structures that they create. RNA secondary structure and GC content of transcripts have been previously implicated with splicing efficiency and exon/intron definition in several organisms including yeast [21]–[25]. However, it is unclear whether the regulatory function of intron-exon junction structure transfers to synthetic contexts as do other sequence motifs, or whether it is lost completely in synthetic contexts as does the ability of introns to splice according to changes in the environment. To verify this we performed a detailed analysis of the correlation between local pre-mRNA folding and GC content and expression levels in our synthetic library. Specifically, we computed the local pre-mRNA folding energy (FE) and GC content profiles of all introns along a sliding window and tested the correlation of these values at each window with the expression levels that we measured. Our analysis demonstrates that introns with unfolded intron-exon junctions tend to exhibit higher expression levels, while introns that induced stronger RNA secondary structures at the intron-exon junction exhibit lower expression levels (Figure 3A). Therefore, we conclude that FE and GC content at intron-exon junctions are significant modulators of synthetic gene expression.

In accordance with previous reports on the effect of RNA secondary structure and GC content on splicing in endogenous genes [21]–[25] our findings provide evidence of intron-exon junctions structure-based regulation in several synthetic contexts. This suggests that junction structure is a modular, transferable regulatory feature that may be useful in the design of synthetic genetic circuits. Moreover, our results suggest principles for an informed design of intron/exon junctions to accurately tune synthetic gene expression systems.

We inserted introns into additional positions within our synthetic gene expression system, collectively creating a gradient of junction folding strengths. These locations along the gene were selected to create intron-exon junctions with either very strong (165 bp from YFP start), strong (original library, 195 bp from start), intermediate (370 bp from start), or weak RNA folds (461 bp from start). Introns were selected to collectively span the expression range measured in the original reporter library and were inserted in each of the 4 positions (Figure 3B). Gene expression measurements of all strains with introns positioned at the strongest fold were decreased compared to the expression of the same introns in the original position that had a weaker fold. Expression data from these 40 unique strains support the notion that strong artificial junction folding strengths negatively regulate gene expression (Figure 3C; p = 8.3e-03). We did not, however, observe increased splicing at junctions with folding energies even weaker than that of the weak fold (position 461) (figure S7). Additionally, since the splice sites in the original location of the complete intron library had relatively strong FE and high GC content this can also explain why these intron reporters displayed lower expression levels compared to an intron-less control. Collectively, our results of varying Intron-exon junctions demonstrate the robustness and wide applicability of intron-exon junction secondary structure design as an efficient tool for splicing mediated control of gene expression in synthetic expression systems, since junction fold strengths both regulate splicing efficiency and are fully transferable between different exonic locations.

### A model of Intronic transcript features enables accurate tuning of gene expression

Deciphering the splicing regulatory “code” [11], [12], [26] is a major ongoing challenge of modern genetics. Hence, from a bio-engineering perspective it would be important to create a set of simple reliable rules for using introns in synthetic systems with accurate, user specified outcomes on gene expression even before the splicing code is completely understood. For this, dictating features of intron splicing in synthetic contexts must be defined and accurate predictions of their effect must be available. To this end, we incorporated the major determinants of intron splicing in synthetic contexts into a model that lays the basic rules and generates accurate and reliable predictions for tuning synthetic gene expression using introns.

We compiled a dataset of intronic features using three independent approaches: first we manually defined simple, intuitive features such as intron length and distances from the branch-point position to both splice sites (See SI for a complete list). Second, we computed various features related to the GC content and local pre-mRNA folding along each intron and intron-exon junctions, as mentioned before (Figure 3A). Finally, we scored each intron for the presence of a sequence motif (table S5).

**Figure 4:**
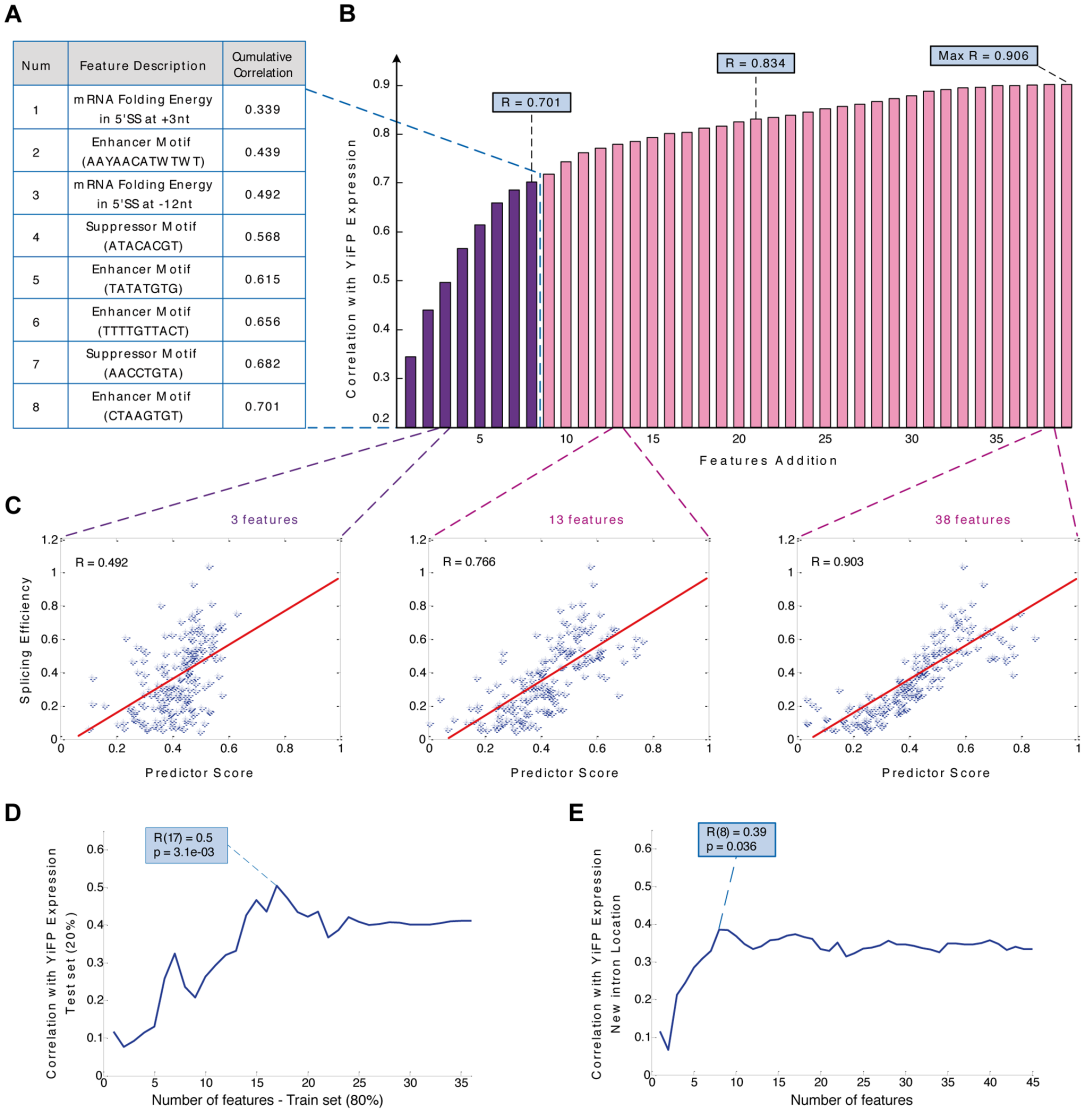
Modeling intron features uncovers design principles and allows the prediction of gene expression in a synthetic system. A) Sequence based predictor of gene expression assembly process: In every iteration the feature contributing the highest correlation to the reporter expression measurements was added. The first eight features and their description are presented. B) Bar diagram of the predictor's cumulative correlation with expression levels of YiFP variants as a function of the number of added features. C) A predictor function based on 3, 13, or 38 features was able to explain 49%, 77% and 90% of gene expression variation, respectively. (for 13 features: p<2.2e-16; empirical p<5e-03); D) Cross validation of the predictor assembly method using training and test sets, with 80% and 20% of introns respectively, demonstrated a predictive power of 50% (for >15 features: 0.37<r<0.5; p<3.6e-02). E) A new predictor assembled using strains with introns inserted to several locations in the YFP maintains 80% of the model's predictive power (r = 0.38; p = 0.036), suggesting that although some of the regulatory splicing information is not located in intronic regions, our methodology is able to predict intron regulation under several exon contexts.

We tested the contribution of each feature in the dataset to gene expression and the top-scoring features validated that RNA structures at intron-exon junctions (r = 0.44; p = 8.21e-06, table S6) as well as several intronic sequence motifs (table S6) were the primary determinants of intron-mediated tuning of synthetic gene expression in this synthetic context. To assess the combined contribution of the various intron features to gene expression levels, we constructed a linear regression function that optimizes combinations of features that accurately account for the empiric expression levels (Materials and methods and table S7). The regression function was built by iteratively adding single features that yield the highest correlation to expression, considering only features with significantly high correlations. We found that local pre-mRNA folding energy at two specific locations spanning the 5′ splice site (+3 nt and −12 nt) as well as several sequence motifs are the principal expression determining features (Figure 4A). Our model yielded correlations of more than 0.7 with the expression measurements using a combination of 8 features, and more than 0.76 using 13 features (Figure 4B & 4C; r = 0.766; p<2.22e-016; empirical p<5e-03; see also Materials and methods and table S7). In contrast, any individual intron feature was only able to explain up to 25% of the observed variation. Despite the detachment of introns from their native context, multiple regulatory mechanisms are still in play “out of context”, emphasizing the significance of analyzing and quantifying multiple intronic features when designing the integration of introns into synthetic expression systems. Notably, our model exhibited similar results when modeling was done for the major subgroups of intron-containing genes (RPGs and non-RPGs, table S7).

To estimate the lower bound of our model's predictive power and account for any potential over-fitting we built new regression functions (including the re-building of the feature database) using a training set composed of 80% of the introns and calculated the correlation between the models' prediction and expression measurements of the remaining 20% (Figure 4D). Our results demonstrated our ability to predict and design the effect of introns on expression in a specific location along a synthetic gene. The bioengineering value of the rules we uncovered and the model we devised as both prediction and design tools for synthetic biology depend, to a large extent, on whether they “transfer” reliably to other exonic contexts. To answer this, we tested our model experimentally on 40 strains placed at four different locations throughout the YFP gene (10 introns at each location, as previously mentioned). We then calculated the correlation between the measured expression and the model predictions using the same set of features for each intron in each location. Surprisingly, despite completely altering the introns exonic context four times, a combination of the eight top intron features maintained 80% of our original model's predictive power (Figure 4E). The ability to maintain predictive power in the face of variable exonic context of introns highlights its gene expression engineering potential, especially in light of the significant and seemingly unpredictable change in gene expression of identical introns in different exonic contexts (Figures S7 & S8).

## Discussion

Synthetic biology aims to create new, finely tuned gene expression systems. A growing repertoire of genetic elements is continuously facilitating the design and construction of more complex synthetic biological systems. In order to enable engineering-level precision in the synthetic control of genetic circuits we must be able to control gene expression at all its levels of regulation – from transcription through splicing and translation. Here we use a combined experimental and computational approach to uncover and formulate rules for using introns in synthetic expression systems. We show that introns can be used to finely control gene expression in a wide dynamic range of expression levels (Figure 1D), and that this tuning can be predicted and designed using a model that integrates several major intronic regulatory determinants (Figure 4B). Our model for assessing the effect of introns on synthetic gene expression based on transcript sequence and structure remained predictive across several exonic contexts (Figure 4E), suggesting that the rules we uncovered reflect genuine rules for intron-mediated tuning of gene expression in synthetic gene expression systems. Our finding that introns lose their environmental responsiveness when placed “out of context” can be utilized in the design of genetic systems tailored to be robust to changes in environmental conditions, in contrast to other genetic elements controlling transcription and translation, which are highly responsive to environmental conditions.

The inability to accurately predict the effect of creating new combinations of genetic elements hinders synthetic biology's ability to streamline the design of novel genetic systems. Our findings and model enables the reliable and robust integration of natural introns, fundamental regulators of gene expression, into synthetic gene expression systems and should be useful for the accurate design and fine tuning of synthetic gene expression systems in general. Finally, our ability to predict the effect of introns through identification of the functional regulatory elements they encode opens the possibility to design synthetic introns with tailored splicing functions in synthetic gene expression systems.

## Materials and Methods

Yeast endogenous intron information and sequences (including GC Content and more) were taken from the Ares Lab database [2] and the Saccharomyces Genome Database (SGD) [27].

### YiFP Library construction

A master strain containing a promoter-less YFP coding sequence (CDS) as well as a Cherry fluorescent protein driven by an independent TEF2 promoter, both inserted at the his3Δ1 locus was used. The master strain was transformed with a library of cassettes, each containing a URA3 selection marker under its own promoter and the YFP splicing reporter with a unique intron.

### YiFP library array

240 YiFP strains were arrayed on SD-URA+NAT agar plates in 384 colony format using a robotic colony arrayer (RoToR, Singer instruments) along with 10 replicates each of various control wells (Text S1).

### Growth and fluorescence measurements

The aforementioned colony arrayer was used to inoculate the library into SD-URA in 384 well microplates (Greiner bio-one, 781162). Following over-night incubation, strains were diluted and cultured in the desired media to a starting O.D_600_ of ∼0.1–0.2 using a robotic liquid handler (Perkin Elmer). A microplate reader (Tecan Infinite M200 monochromator) was used to measure growth (Absorbance at 600 nm), mCherry (E.x. 570 E.m. 630) and YFP expression (E.x. 500 E.m. 540).

### Single cell fluorescence measurements

Single cell fluorescence measurements were performed using an automated microscope system as described in Cohen and Schuldiner, Methods Mol. Biol. 781, 127–59 (2011). Briefly, strains were cultured over-night and diluted in the same manner as in the microplate reader measurements. Following an incubation of four hours in 30°c in a shaking incubator (LiCONiC Instruments), cells were then transferred onto glass bottom 384-well microscope plates (Matrical Bioscience) coated with Concanavalin A (Sigma-Aldrich). The microscope plates were conveyed to an automated inverted fluorescent microscopic ScanR system (Olympus), equipped with a cooled CCD camera. Images were acquired using a 60× air lens using YFP (E.x. 490/20 nm, E.m 535/50 nm), mCherry (E.x. 572/35 nm, E.m 632/60 nm), and bright-field channels. After acquisition images were analyzed using the ScanR Analysis software (Olympus), and single cells were recognized based on the mCherry channel. Measures of cell size, shape and fluorescent signals were extracted. The top and bottom scoring single cells in terms of cell size and shape within each strain were gated out of further analysis to ensure homogenous and correct cell recognition, yielding a mean of 435±164 cells analyzed per strain (minimum of 69 cells).

### mRNA quantification of YFP reporter

mRNA level measurements were performed using quantitative real-time PCR (qPCR). Strains were grown to mid-log and RNA purification was performed using the MasterPure yeast RNA purification kit (Epicentre). cDNA was generated using the SuperScript III First Strand Synthesis kit (Invitrogen). qPCRs were performed in a StepOnePlus Real-Time PCR system (Applied Biosystems) using Fast SYBR Green Master Mix, with ACT1 gene as reference. Relative expression results (RQ) were calculated using the StepOne software (figure S4).

### Motif mutagenesis

Intron transformation cassettes were ligated into the pGEM-T Easy vector (Promega). Mutated transformation cassette was transformed into the master strain as previously described and positive clones were verified by PCR and sequencing.

### Expression data analysis

YFP and O.D. information were filtered using Butterworth IIR Low Pass Filter (LPF) with normalized cutoff frequency of 0.15. Medium (O.D.) and background (no YFP) noise were subtracted, and YiFP or O.D. values were ignored if close to zero or negative (replaced with NaN). The normalized unbiased expression level was calculated using the following equation:
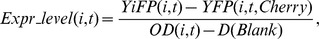
where *i* is the strain number, *t* is the time, *YFP (i, t, Cherry)* is the closet strain on plate without YFP and *OD (Blank)* is the O.D. level of a control well with medium only. YFP-wt strains expression calculations were done in the same manner.

The time Interval threshold was set to be 6 hours, after which an Intron cannot be considered as spliced. In addition, introns with more than half NaN values were considered to be Not Spliced. The rest of the introns were examined based on self-crossing Signal-to-Noise Ratio (SNR) according to the following equation:
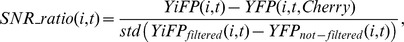
where *YFP not-filtered(I,t)* an*d YFP filtered(I,t)* are raw and filtered YFP data respectively and *std* is a standard deviation. Introns were termed *Spliced* for SNR_ratio higher than 5 in the time interval of the first 6 hours.

The experiments were done in duplicates. The expression levels of *n* repeats were incorporated in the following manner: The average expression level was calculated for each duplicate. The joint expression matrix was obtained according to the following equation:
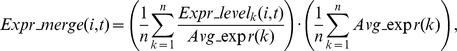
where *k* is the strain number and *n = 4* is the number of duplicates. The maximal expression level merging was done in the same manner. Introns that were considered to be spliced in the majority of the duplicates (3 or more when *n = 4*), were considered to be spliced in the incorporated database.

Splicing efficiency and maximal splicing efficiency were calculated using the following equations respectively:
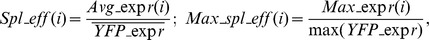
where *i* is the strain number.

### Computational and statistical analysis

RNA secondary structure and folding energy predictions were done using *rnafold* (Vienna) function [28]. 2D distance calculations were done using RNA secondary structure predictions and the *Dijkstra* minimum path algorithm [29].

*De-Novo* Motifs & enriched sequences were identified using the HOMER (Hyper-geometric Optimization of Motif Enrichment) tool [30]. Only significant motifs were later used as expression predictors. Motifs distribution analysis was performed by generating a set of random motifs using internal motif permutation tests that preserve original motif properties. The location and significance level of the random motifs were calculated (Table S4).

Calculation of distance between motifs was done by comparing their probability matrices using the following formulation:
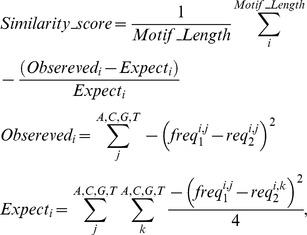
where freq_1_ and freq_2_ are the matrices for motif_1_ and motif_2_, respectively. Empirically significant motifs with similarity score higher than 0.6 were merged.

### Linear regressor assembly

Prediction features were put into a linear regressor to assemble an expression predictor and a feature assembly list was calculated. Accumulation of features was done using greedy algorithm. In each feature assembly iteration k, spearman correlation was calculated. The adjusted correlation, which considers the number of features, value was calculated according to the following formula:
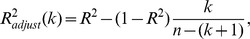
where *n* is the number of measurement features, and R is the Spearman correlation in the *k-th* iteration. The robustness of the predicator results was validated using several statistical methods including permutation tests and cross validation analysis. See Text S1 for additional methods information.

## Supporting Information

Figure S1 The genomic content of the YiFP library at the his3Δ1 locus. Each strain in the library contains (in order from 5′ to 3′) an mCherry fluorescent protein, a URA3 selection marker, RPS28A promoter, 195 bp of YFP (yEVenus), an intron, the remaining 523 bp of YFP, and a NAT selection marker. The sequence that was introduced into the master strain is marked in green as “Transformation cassette.”
(JPG)

Figure S2 4 duplications of the synthetic YiFP expression level results in time for Spliced intron genes using normal growth conditions (SD complete media).
(JPG)

Figure S3 Merged representation of the synthetic YiFP expression levels results in time for all introns (top) and Spliced intron (bottom) using normal growth conditions (SD complete media).
(JPG)

Figure S4 YFP reporter fluorescence measurements reflect mRNA abundance. Splicing efficiency, a relative quantification of YFP fluorescence in YiFP strains compared to YFP-WT, is highly correlated to relative YFP mRNA abundance as calculated from qPCR (R2 = 0.975; p = 2.3e-04).
(JPG)

Figure S5 Single cell expression analysis confirms splicing efficiency index and enables the assessment of cell-to-cell variability. Representative images of five YiFP strains are shown along with their splicing efficiency score (based on plate-reader measurements), and single cell analysis of mean YFP and its coefficient of variation (CV). NOG2 was found to have a significantly higher CV than expected (see figure 1F).
(JPG)

Figure S6 Motifs location distribution analyses - Enrichment of intronic motifs (top, blue line) compared to randomized/permutated introns (yellow line) is presented in respect to distance from 5′ or 3′ SS (left and right respectively). Motifs difference is presented in the bottom. Positions significantly enriched or deprived of motifs are marked in red and green respectively: A) all introns; B) non-spliced introns; C) spliced introns; D) spliced ribosomal introns; E) spliced non-ribosomal introns.
(JPG)

Figure S7 All new location strains detailed expression levels over time. For each strain, the following information is presented: introns-less YFP expression, original YiFP expression (location 195), strong folding expression (location 165), weak folding expression (location 461) and intermediate folding expression (location 370).
(JPG)

Figure S8 Exonic context dramatically affects the splicing of introns. Top left – the expression level (equivalent to splicing efficiency) of 10 introns in one location along the YFP (195 nt from the YFP's ATG) are plotted against the same 10 introns expression in duplicate experiment as a control for reproducibility (correlation of 0.9901, p = 4.2133e-08) showing. Conversely, the correlation between expression measurements of the same 10 introns at different exonic locations drops significantly. Specifically, on the bottom left panel we plot the same 10 introns expression in location 195 (Y axis) against their expression in location 165 (165 nt from ATG, Strong FE) and show that intron expression is altered significantly upon displacement to other exonic locations (r = 0.6780, p = 0.0312). The same analysis was performed with similar results for the two other locations, 10 introns each (461 nt from ATG – bottom right panel, Maximal FE, r = 0.4593, p = 0.182 (N/S) and 370 nt from ATG, Intermediate FE, r = 0.8435, p = 2.1637e-03).
(JPG)

Figure S9 Distribution of growth rates. A histogram of the distribution of growth rates for all YiFP library strains is shown for the three environmental condition tested (AA starvation, KCL and Rapamycin).
(TIF)

Figure S10 Robustness of splicing efficiency for all three conditions (AA starvation, KCL and Rapamycin) for all YiFP library strains is shown on a log-log plot (in contrast to the linear plotting in figure 1G). Top 10 strain with the highest variation from the linear regression line are named on the graph for each condition (see also Table S9).
(TIF)

Table S1 Intron-reporter expression database. This table summarizes the YiFP expression analysis of the data generated by the micro-plate reader. The splicing efficiency is shown for all strains (spliced as well as non-spliced) including all the replications and the merged information.
(XLSX)

Table S2 Conditions experiments raw data. The experimental conditions raw data contains all the different duplication information and internal condition splicing efficiency correlation results and p-values. The growth levels of the Cherry (No-YFP) and YiFP strains are presented as well as the YiFP strains' growth level standard deviation.
(XLSX)

Table S3 Conditions summary. This table summarizes the various experimental conditions. For each condition the Cherry and YFP average growth rate and internal splicing efficiency correlation are presented. The condition current correlations (Pearson) and p-value with the other condition are presented afterwards.
(XLSX)

Table S4 **Motif location analysis.** This table summarizes the frequency and position of appearance of the identified motifs along the introns, specified as distances from the 5′ and 3′ of the introns. For each of the motifs the table also describes its minimal and maximal scores, as well as which clans each motif belongs to.
(XLSX)

Table S5 Top motifs list. This table contains the motifs that have the highest correlation (Spearman) with the measured average and maximal expression levels. The p-values and the motif clan number are also presented. All the motifs have passed FDR.
(XLSX)

Table S6 Feature summary. This table summarizes the all features that were constructed and their correlation (Spearman) with the measured average and maximal expression levels. Each feature correlation p-value and empirical p-value is also presented. Some of the features are a bundle of a singular sub-features (e.g. FE in different intronic locations); in this case the bundle best location is also presented. The spliced Introns are divided into 2 subgroups: ribosomal and non-ribosomal.
(XLSX)

Table S7 Regressor summary. This table summarizes the linear predictor (regressor) assembly buildup for the average and maximal expression levels. For each stage the additional feature and current correlation (Spearman) are presented, as well as the p-value and empirical p-value. The spliced Introns are divided into 2 subgroups: ribosomal and non-ribosomal.
(XLSX)

Table S8 Growth rate outliers. This table shows intron strain growth rate outliers strains that exhibit a growth rates that are more than two standard deviations from the mean, per condition (showed for Rapamycin, AA starvation and KCL).
(XLSX)

Table S9 Splicing efficiency outliers. This table shows the distance of each intron strains splicing efficiency from the linear regression splicing efficiency line (figure 1G and figure S9) for all three conditions (Rapamycin, AA starvation and KCL). For each condition, the top 10 strains with the highest distance from the regression line are marked here and also named in figure S9.
(XLSX)

Text S1 *A.* Description of the methods used for the construction and transformations of the YiFP libraries. B. Description of the methods used for the quantification and characterization of YiFP gene expression. C. Description of the computational methods used for the analysis of intron sequence motifs. D. Description of the computational methods used for the analysis of intron features, building a regressor function and assessing their statistical significance.
(DOCX)
