# Supplementary figures and images for: Accurate, Model-Based Tuning of Synthetic Gene Expression Using Introns in S. cerevisiae
Source: PLoS Genet. 2014 Jun 26;10(6):e1004407. doi: 10.1371/journal.pgen.1004407 (PMC4072511; doi:10.1371/journal.pgen.1004407)

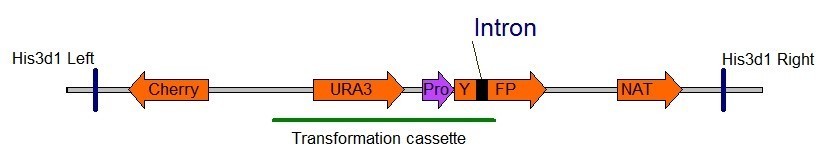

Supplement: Figure S1 — The genomic content of the YiFP library at the his3Δ1 locus. Each strain in the library contains (in order from 5′ to 3′) an mCherry fluorescent protein, a URA3 selection marker, RPS28A promoter, 195 bp of YFP (yEVenus), an intron, the remaining 523 bp of YFP, and a NAT selection marker. The sequence that was introduced into the master strain is marked in green as “Transformation cassette.” (JPG) [file pgen.1004407.s001.jpg]

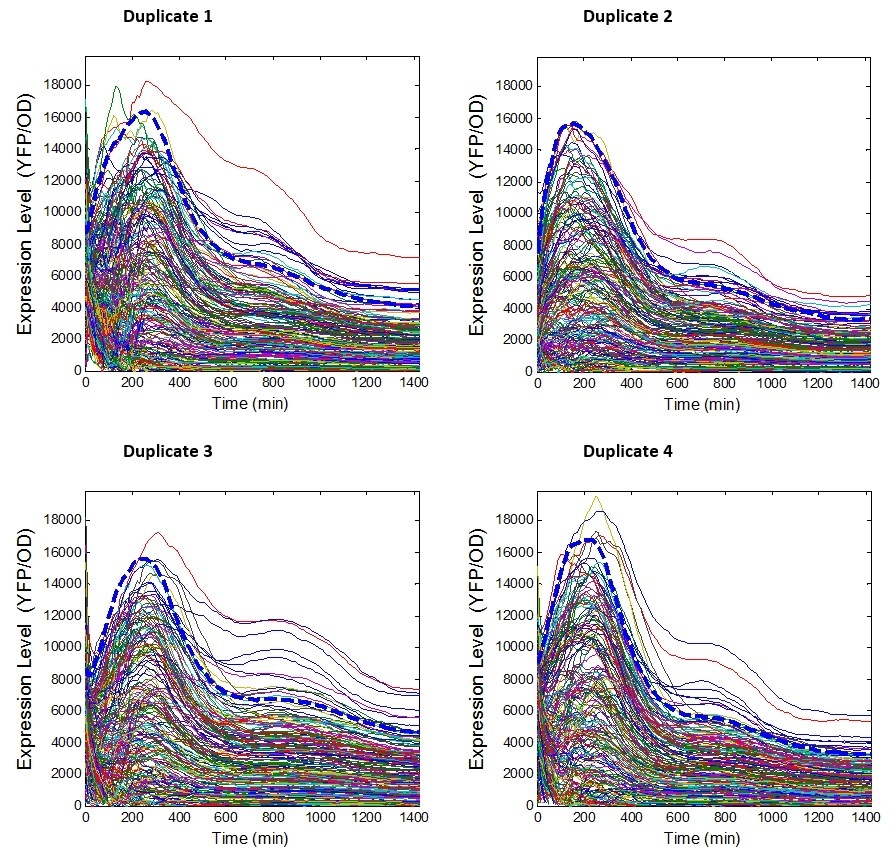

Supplement: Figure S2 — 4 duplications of the synthetic YiFP expression level results in time for Spliced intron genes using normal growth conditions (SD complete media). (JPG) [file pgen.1004407.s002.jpg]

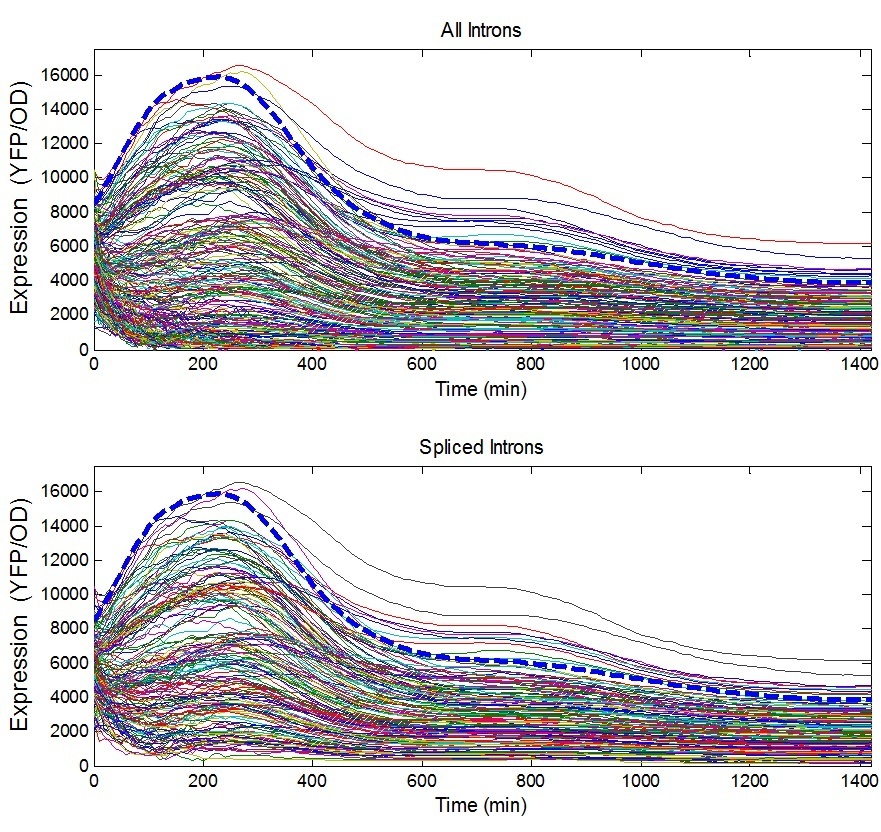

Supplement: Figure S3 — Merged representation of the synthetic YiFP expression levels results in time for all introns (top) and Spliced intron (bottom) using normal growth conditions (SD complete media). (JPG) [file pgen.1004407.s003.jpg]

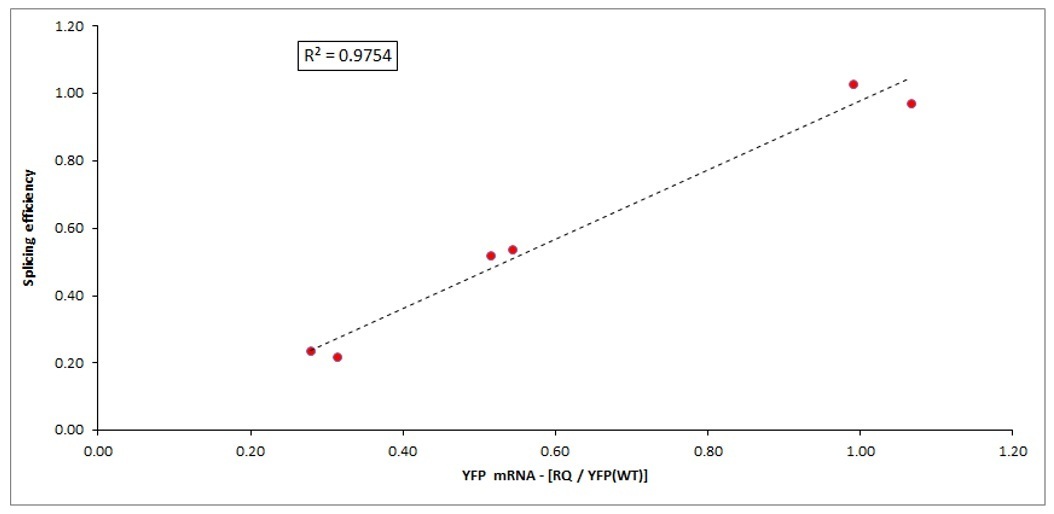

Supplement: Figure S4 — YFP reporter fluorescence measurements reflect mRNA abundance. Splicing efficiency, a relative quantification of YFP fluorescence in YiFP strains compared to YFP-WT, is highly correlated to relative YFP mRNA abundance as calculated from qPCR (R2 = 0.975; p = 2.3e-04). (JPG) [file pgen.1004407.s004.jpg]

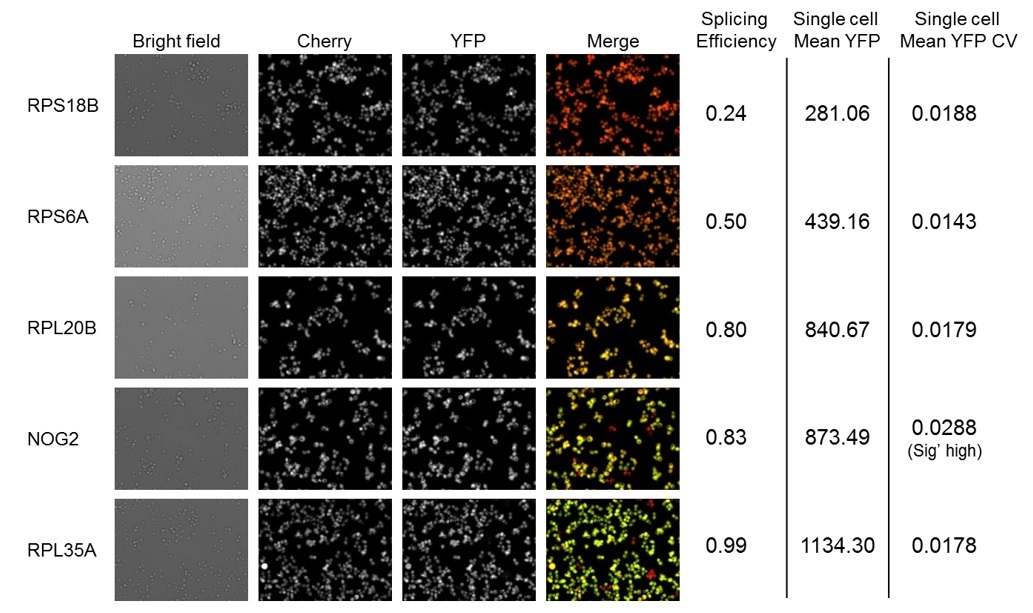

Supplement: Figure S5 — Single cell expression analysis confirms splicing efficiency index and enables the assessment of cell-to-cell variability. Representative images of five YiFP strains are shown along with their splicing efficiency score (based on plate-reader measurements), and single cell analysis of mean YFP and its coefficient of variation (CV). NOG2 was found to have a significantly higher CV than expected (see figure 1F). (JPG) [file pgen.1004407.s005.jpg]

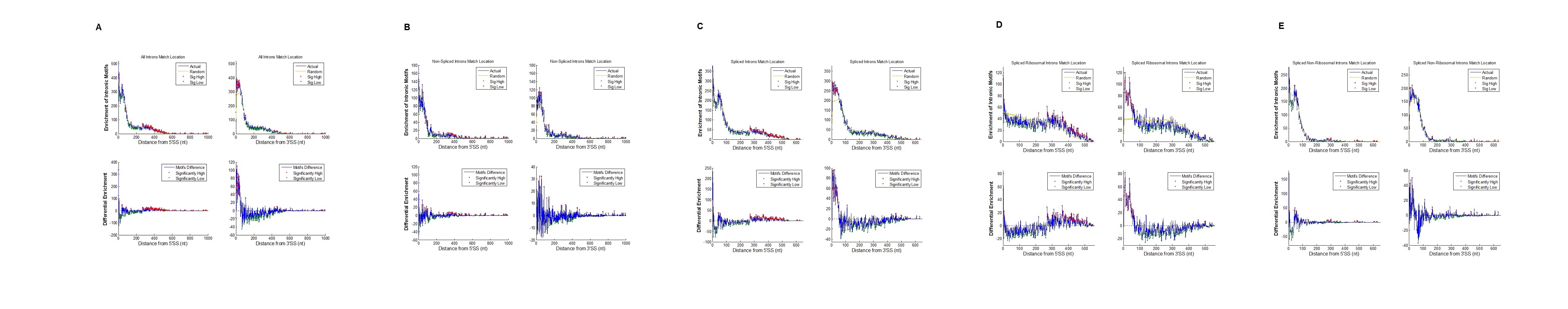

Supplement: Figure S6 — Motifs location distribution analyses - Enrichment of intronic motifs (top, blue line) compared to randomized/permutated introns (yellow line) is presented in respect to distance from 5′ or 3′ SS (left and right respectively). Motifs difference is presented in the bottom. Positions significantly enriched or deprived of motifs are marked in red and green respectively: A) all introns; B) non-spliced introns; C) spliced introns; D) spliced ribosomal introns; E) spliced non-ribosomal introns. (JPG) [file pgen.1004407.s006.jpg]

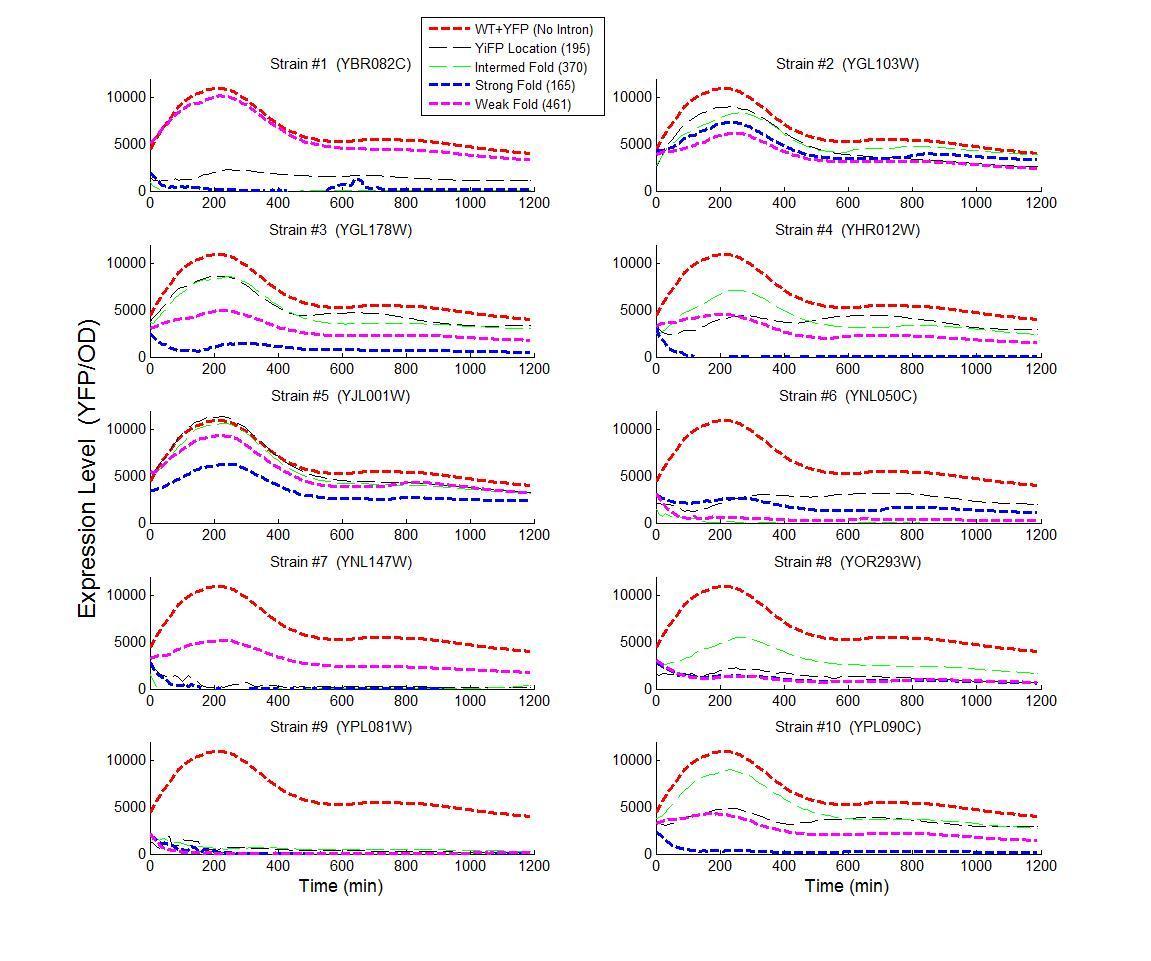

Supplement: Figure S7 — All new location strains detailed expression levels over time. For each strain, the following information is presented: introns-less YFP expression, original YiFP expression (location 195), strong folding expression (location 165), weak folding expression (location 461) and intermediate folding expression (location 370). (JPG) [file pgen.1004407.s007.jpg]

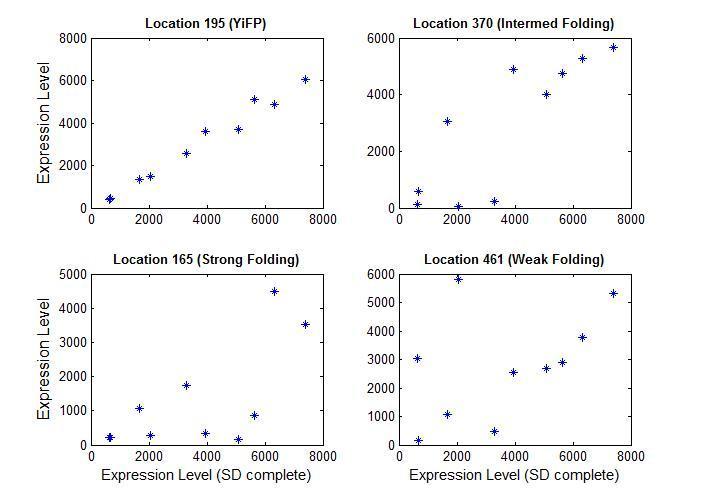

Supplement: Figure S8 — Exonic context dramatically affects the splicing of introns. Top left – the expression level (equivalent to splicing efficiency) of 10 introns in one location along the YFP (195 nt from the YFP's ATG) are plotted against the same 10 introns expression in duplicate experiment as a control for reproducibility (correlation of 0.9901, p = 4.2133e-08) showing. Conversely, the correlation between expression measurements of the same 10 introns at different exonic locations drops significantly. Specifically, on the bottom left panel we plot the same 10 introns expression in location 195 (Y axis) against their expression in location 165 (165 nt from ATG, Strong FE) and show that intron expression is altered significantly upon displacement to other exonic locations (r = 0.6780, p = 0.0312). The same analysis was performed with similar results for the two other locations, 10 introns each (461 nt from ATG – bottom right panel, Maximal FE, r = 0.4593, p = 0.182 (N/S) and 370 nt from ATG, Intermediate FE, r = 0.8435, p = 2.1637e-03). (JPG) [file pgen.1004407.s008.jpg]

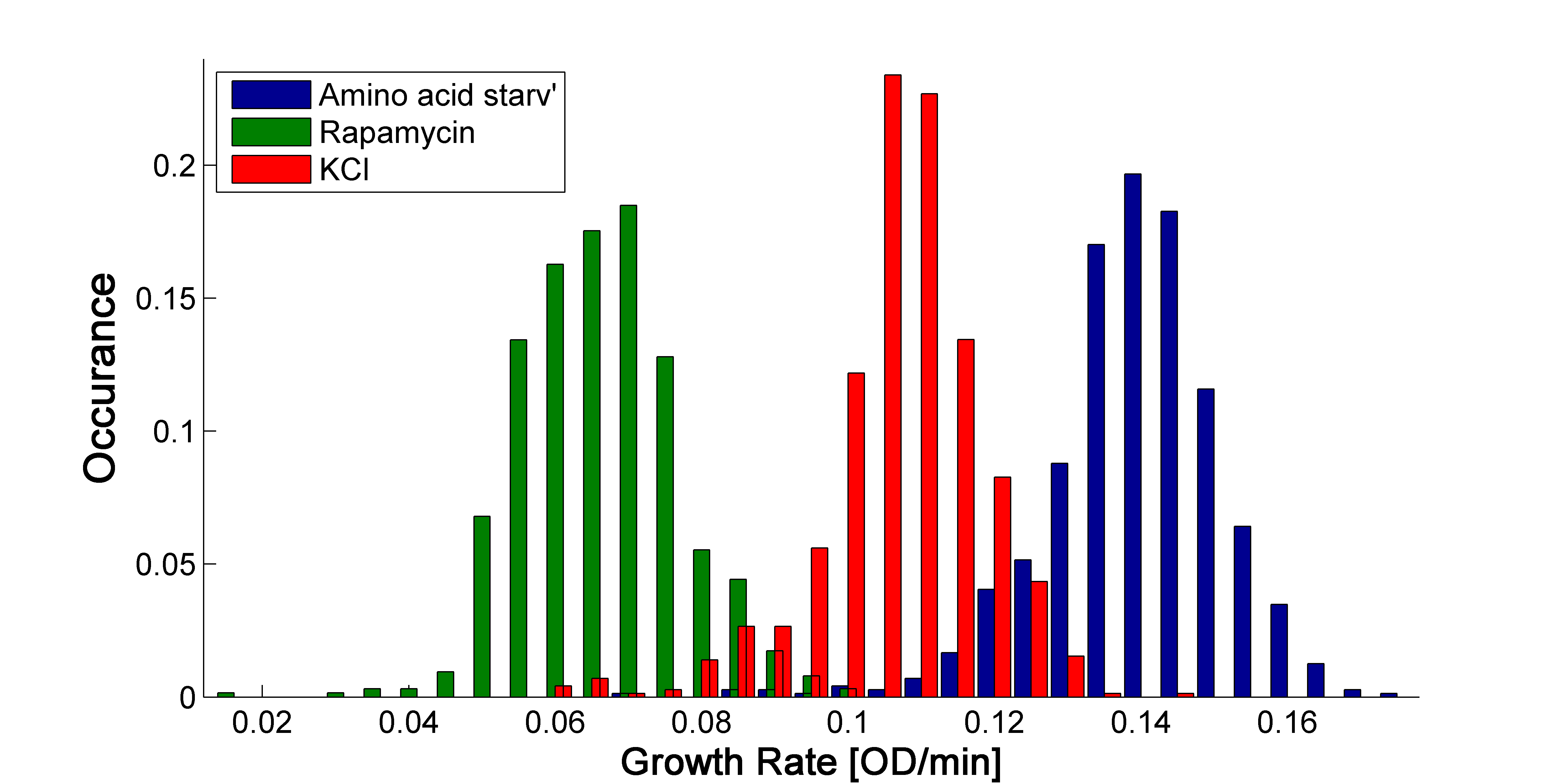

Supplement: Figure S9 — Distribution of growth rates. A histogram of the distribution of growth rates for all YiFP library strains is shown for the three environmental condition tested (AA starvation, KCL and Rapamycin). (TIF) [file pgen.1004407.s009.tif]

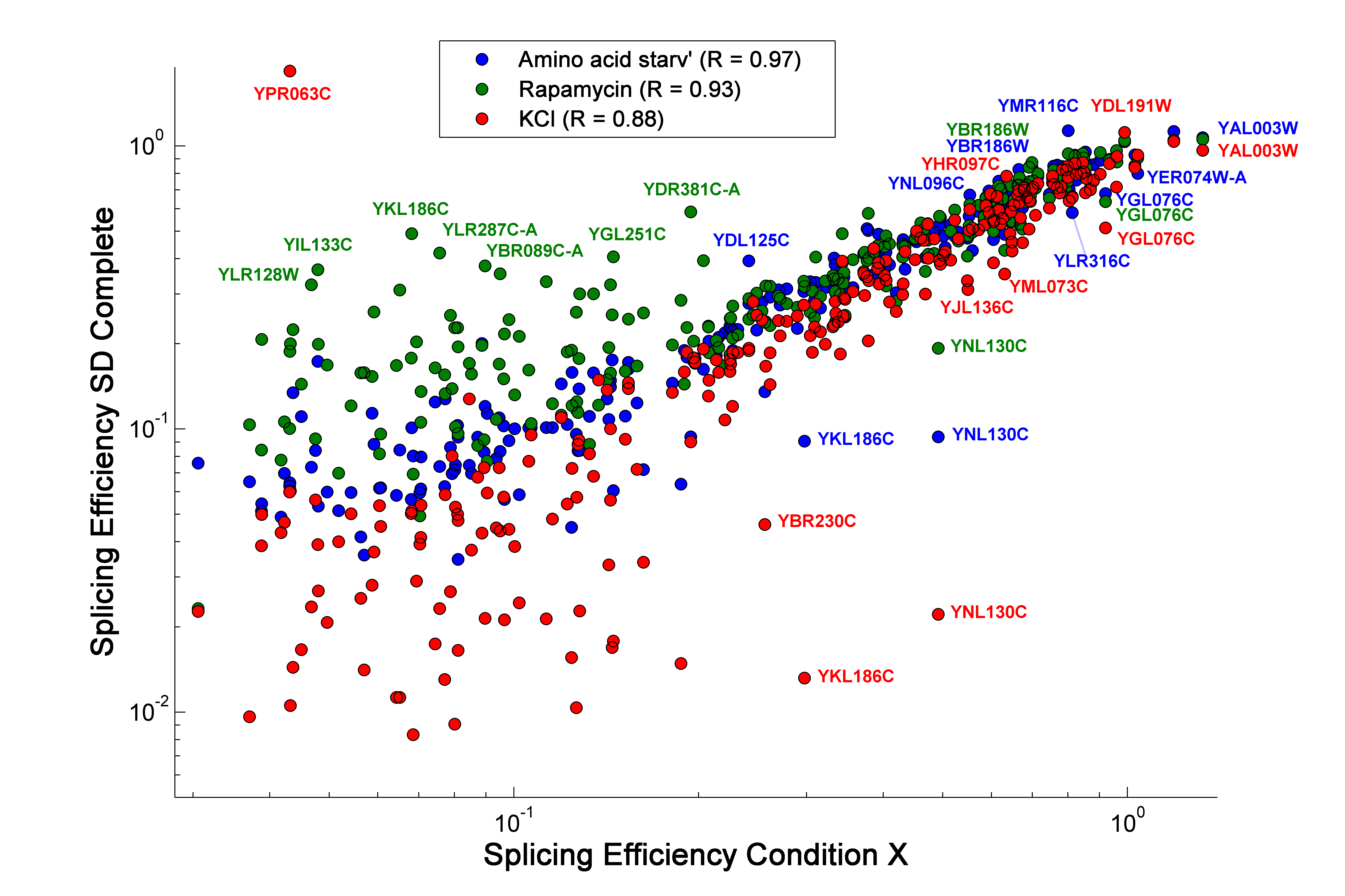

Supplement: Figure S10 — Robustness of splicing efficiency for all three conditions (AA starvation, KCL and Rapamycin) for all YiFP library strains is shown on a log-log plot (in contrast to the linear plotting in figure 1G). Top 10 strain with the highest variation from the linear regression line are named on the graph for each condition (see also Table S9). (TIF) [file pgen.1004407.s010.tif]
