# Supplementary material for: Accurate, Model-Based Tuning of Synthetic Gene Expression Using Introns in S. cerevisiae
Source: PLoS Genet. 2014 Jun 26;10(6):e1004407. doi: 10.1371/journal.pgen.1004407 (PMC4072511; doi:10.1371/journal.pgen.1004407)
Supplement: Text S1 — A. Description of the methods used for the construction and transformations of the YiFP libraries. B. Description of the methods used for the quantification and characterization of YiFP gene expression. C. Description of the computational methods used for the analysis of intron sequence motifs. D. Description of the computational methods used for the analysis of intron features, building a regressor function and assessing their statistical significance. (DOCX) [file pgen.1004407.s020.docx]

**Text S1**

***YiFP Library construction overview***

In order to create a synthetic library of yeast with introns inserted into a yellow fluorescent protein (YFP) we utilized a master strain already containing a promoter-less YFP coding sequence (CDS) as well as a Cherry fluorescent proteins driven by an independent *TEF2* promoter, both inserted at the *his3∆1* locus. This strain was transformed with a library of cassettes, each containing a promoter followed by the 5’ of the YFP, connected to a different *S. cerevisiae* intron, and a URA3 selection marker under its own promoter.

***Design of primers for yeast intron amplification***

Primer pairs were made to amplify 240 natural introns of *S. cerevisiae*. Each primer was designed to (A) complement the 5'- or 3'- end of a specific intron (optimal temperature was set to 60°c, mean of ≅29 nucleotides (nt)), (B) contain a 35 nt overhangs of YFP (yEVenus variant) CDS, upstream or downstream (respectively) to nucleotide 195 in the YFP sequence, *i.e.* the intron insertion location. The common YFP overhang sequences assured identical location of all introns within the YFP, and served as the basis for later synthesis steps. Primers were all synthesized by Sigma-Aldrich (Rehovot, Israel), using standard (desalt) purification. All 5'-primers were phosphorylated using T4 polynucleotide kinase (PNK, New England Biolabs, M0201) according to later synthesis steps protocol.

***PCR amplifications of Introns***

Intron fragments were amplified using the previously described primers on genomic sequence templates. Template sequences were made from colonies of bacterial strains of the MoBY {Formatting Citation}or Tiling genomic yeast libraries^1-2^. The amplification was verified using gel electrophoresis. All PCR reactions were performed in 96 well PCR micro-plates, using KOD Hot Start DNA Polymerase (Novagen, 71086-3) according to protocol. Elongation times were set to be sufficient for amplification of the longest intron in each plate.

***Transformation cassette library construction using “Y-operation”***

Y-operation is a procedure that allows the precise connection of two DNA fragments ^3-5^. The DNA fragments are amplified by primers that include overhangs to produce an overlap between the two resulting double stranded DNA (dsDNA) molecules. The primers' overhangs are phosphorylated at their 5' ends. Lambda (λ) exonuclease is used to digest the phosphorylated strands resulting in two overlapping single stranded DNA (ssDNA) molecules. The two ssDNA molecules are mixed and an elongation reaction by a polymerase is facilitated by reciprocal priming of complementary overhangs. The output dsDNA molecule is a concatenation of the original two fragments. See the complete protocol elswhere^12^.

Using Y-operation, the intron DNA fragment library was joint to a common DNA fragment containing (1) A homologous sequence for targeting recombination into the *his3∆1* locus, (2) A URA3 selection marker (with its own promoter and terminator), (3) The promoter of *S. cerevisiae* gene *RPS28A*, and (4) The 5' end 195 nt of the YFP CDS (see Figure S1). The *RPS28A* promoter was chosen as it confers high expression level of the YFP fluorescent protein following it which remains stable under various conditions^5^. The end result of the Y operation was a library of transformation cassettes (each including the URA3 selection marker), a *RPS28A* promoter, 230 nt of YFP CDS, and a different intron inserted at the 196^th^ nt of the YFP (see Figure S1, denoted as “Transformation cassette”).

***The transformation “master strain”***

The library transformation master strain was generously provided by Eran Segal. The master strain was created by integrating into the yeast genome a cassette expressing the fluorescent mCherry under a TEF2 promoter and a promoter-less YFP, followed by a NAT (Nourseothricin) resistance marker under its own promoter. The entire sequence was inserted into the his3∆1 locus of strain Y8205 (A strain derived from *S. cerevisiae* S288C, BY4741, mat alpha, Charlie Boone lab).

***High-throughput transformations***

The transformation cassettes were transformed into the master strain using high throughput transformations. A culture of the master strain was grown in rich medium to mid log (O.D_600_ = 0.75). The culture was washed twice by (1) centrifugation for 5 minutes at 2500 RCF at room temperature, (2) removal of the supernatant, and (3) re-suspension of the cell pellet with 0.1M Lithium Acetate (LiAc). The cell culture was finally concentrated by a factor of 100 in 0.1M LiAc. A transformation mix was prepared containing the following amount per each transformation: 100 µl of 50% (w/v) filter sterilized PEG 3350, 15 µl of 1M filter sterilized LiAc, 20 µl of 2mg/ml boiled carrier DNA, 18 µl of DMSO, and 15 µl of the concentrated cell culture. At this point 168 µl of the transformation mix were transfered into each PCR microplate tube already containing 30 µl of the PCR amplyfied transformation cassettes. The cells were then incubated using PCR block for 30 minutes at 30°C, followed by 15 minutes at 42°C. The PCR block lid was kept at 30˚c throughout the entire reaction. The PCR microplates were then centrifuged for 1 minute at 2500 RCF at room temperature and the supernatant was discarded. Cells were re-suspended in 50 µl of synthetic media with 2% dextrose lacking Uracil (SD-URA), transferred to agar plates containing SD-URA selective media, and incubated at 30°c for 3-4 days. Transformed colonies were hand-picked and patched on SD-URA+NAT (Werner Bioagents) agar plates. Correct transformation was verified in all strains by PCR amplification from the yeast's genome, using primers flanking the intron insertion site and gel electrophoresis. Sample of strains were additionally verified by sequencing of the inserted introns and the region flanking the insertion site; no mutations were found. In total, 240 strains were successfully verified, each containing a different *S. cerevisiae* intron inserted in the YFP CDS, named YiFP strains.

***Inserting introns into different contexts***

Transformation cassettes were made and transformed into the master strain as previously described. However, the introns were inserted in these cases after either nucleotide 165, 370, and 461 of the YFP CDS. All clones were verified and measured as previously described.

***YiFP library array***

The 240 YiFP strains were arrayed on agar plates with SD-URA+NAT media, in 384 colony format using a robotic colony arrayer (RoToR, Singer instruments), along with control strains which included strains without an intron to serve as a reference (YFP-WT), as well as strains that express only mCherry (No-YFP). Each control strain has 10 replicates in the library plate. In addition, 10 locations were kept empty to be used as blank wells (medium control).

**B*.***

***Growth and fluorescence measurements***

In order to measure growth and fluorescent protein expression, the Singer colony arrayer was used to inoculate all colonies of the library into 100 µl of SD-URA media in a 384 well growth microplate (Greiner bio-one, 781162). Following 15 hours of incubation, 5 µl of the yeast cultures were diluted into 95 µl of SD complete media in 384 well growth microplate, to reach a starting O.D_600_ of ~0.1-0.2, using a robotic liquid handler (Perkin Elmer). To examine stress conditions known to affect splicing, cultures were diluted in a similar manner to the following media: Amino acid starvation: Yeast nitrogen base without amino acids and ammonium sulfate+ 2% Glucose +HIS+ MET. Rapamycin: SD complete + 200ng/ml Rapamycin. KCl: SD complete + 1M KCl. A microplate reader (Neotec Infinite M200 monochromator) was then set to measure the following parameters in cycles of 10 minutes: Cell growth (as extracted from absorbance at 600nm); mCherry expression (excitation wavelength (E.x.) 570, Emission wavelength (E.m.) 630); and YFP expression (E.x. 500 E.m. 540). Cultures were shaken in between measurements. All incubation and measurements were done in 30°c.

***Variability of growth Rate***

In order to verify that the intron in the YiFP reporter does not impose a significant effect on cell growth, we performed an analysis comparing the variability in growth rate of all 240 YiFP strains to that measured in 10 independent repeats of the same strain, for the two control strains (No-YFP and YFP-WT). The analysis was done separately for each of the four independent repeats of library measurements. Growth rates were calculated according to the OD slopes in the exponential growth phase, using a polynomial curve fitting function to find the OD curve coefficients. The analysis (supplementary table S2) showed that the mean growth rate for YiFP strains (0.1692 OD/minute) is highly similar to that of No-YFP (0.1694) and YFP-WT (0.1721).

***mRNA quantification of YFP reporter***

In order to verify that the fluorescence measurements reflect the reporter mRNA levels we performed quantitative real-time PCR (qPCR) measurements to assess the relative YFP mRNA abundance in six YiFP strains, compared to the YFP-wt strain (no intron). The following six strains were picked from the YiFP library to span both expression levels (splicing efficiency 0.2-0.93) and intron length (95-511 bp): YGL103W, YJL001W, YPL090C, YHR012W, YOR293W, and YBR082C. All strains were grown to mid-log (O.D_600_=0.6-0.7) and RNA purification was performed using the MasterPure™ yeast RNA purification kit by Epicentre. Next, cDNA was created using the SuperScript® III First Strand Synthesis kit by Invitrogen (using random hexamers as primers). qPCRs were performed in a StepOnePlus™ Real-Time PCR system (Applied Biosystems) using Fast SYBR® Green Master Mix according to its protocol with primers for YFP (forward primer spanning the exon-intron junction of the YiFP) and ACT1 as a reference. In addition, no-template (water), and no-reverse transcriptase (RT) reactions were performed to control for DNA contaminations. Relative YFP levels for all YiFP strains compared to the YFP-wt strain (RQ) were calculated using the StepOne software (Figure S4).

***Expression data processing***

The expression data measured was analyzed and normalized in the following manner:

1. Plate raw data was examined: Plate data contains O.D. (Optical Density), YFP fluorescent levels and control information were taken from an excel file generated by the micro-plate reader.
2. The data was parsed according to the strains information (YiFP, control strains), and results were integrated with pre-constructed intron database.
3. YFP and O.D. information were filtered using Butterworth IIR Low Pass Filter (LPF) with normalized cutoff frequency of 0.15.
4. Medium (O.D. of blank wells) and background YFP fluorescence noise (no YFP strain) were subtracted, and YiFP or O.D. values close to zero or negative were masked.
5. The normalized unbiased expression level matrix was calculate using the following equation: $Expr\_level\left( i,t \right)=\frac{YiFP\left( i,t \right)-YFP(i,t, Cherry)}{OD\left( i,t \right)-OD(Blank)}$ , where *i* is the strain number, *t* is the time, *YFP (i, t, Cherry)* is the closet strain on plate without YFP and *OD (Blank)* is the O.D. level of a control well with medium only. YFP wild type strains expression calculations were done in the same manner.
6. The time interval threshold was set to be 6 hours, after which an Intron cannot be considered as spliced.
7. The rest of the introns were examined based on self-crossing Signal-to-Noise Ratio (SNR) according to the following equation:

$$SNR\_ratio\left( i,t \right)=\frac{YiFP\left( i,t \right)-YiFP(i,t, Cherry)}{std(YiFP_{filterd}(i,t)- YiFP_{not\_filterd}(i.t))}$$

, where *YFP not-filtered(I,t)* an*d YFP filtered(I,t)* are raw and filleted YFP data respectively and *std* is a standard deviation. Introns were termed ***Spliced*** for SNR ratio higher than 5 in the time interval of the first 6 hours.

1. The experiments were done in duplicates. The expression levels of *n* repeats were incorporated in the following manner:
   1. The average expression level over time was calculated for each duplicate.
   2. The joint expression matrix was obtained according to the following equation:

$$Expr\_merge\left( i,t \right)=\left( \frac{1}{n}\sum_{k=1}^{n} \frac{Exp{r\_level}_{k}\left( i,t \right)}{Avg\_expr\left( k \right)} \right)\cdot\left( \frac{1}{n}\sum_{k=1}^{n} Avg\_expr\left( k \right) \right)$$

, where *k* is the strain number and *n = 3 or 4* is the number of duplicates.

- 1. The merging of another parameter, the maximal expression level was done in the same manner.
  2. Introns that were considered to be spliced in the majority of the duplicates (3 or more when *n = 4*), were considered to be spliced in the incorporated database.

1. Related statistics were extracted and analyzed (maximal and average expression levels, splicing efficiency etc.).
2. Splicing efficiency and maximal splicing efficiency were calculated using the following equations respectively:

$Spl\_eff(i)=\frac{Avg\_expr(i)}{\bar{YFP\_expr}}$ ; $Max\_spl\_eff(i)=\frac{Max\_expr(i)}{max\left( YFP\_expr \right)}$

, where *i* is the strain number.

***Duplicated Correlation Values***

0.93 < Avg_Corr < 0.96 (Same as Splicing Efficiency)

0.63 < Max_Corr < 0.83 (Same as Maximal Splicing Efficiency)

***The merged expression results***

During the incorporation, 4 outliner introns were excluded (YCR097W (2nd intron), YIL148W, YKL006W, YLR275W) due to strain growth issues, and were not considered as part of the library information. A total of 240 introns were analyzed. A total of 178 introns were termed Spliced. In particular, a total of 86 Ribosomal introns were termed Rib-Spliced and a total of 92 Non-Ribosomal introns were termed NoRib-Spliced.

C.

***Identifying motifs enrichment***

We binned introns to either low or high expression groups and looked for sequence motifs enriched in each group using the HOMER tool (Hyper-geometric Optimization of Motif Enrichment) (Heinz et al. 2010). Notably, this analysis eliminates the canonical splice and branch sites as possible features as they are found in all introns, and are extremely conserved in S. cerevisiae. We performed these comparisons for all introns, sub-groups of RPGs and non-RPGs, and with various binning of expression levels (supplementary information). After false discovery rate (FDR) filtering and merging of resembling motifs, our analysis shows intronic motifs (Figure 2 and supplementary tables S4, S5 & S6; p < 6.35e-03 for all cases) that are predicted ISEs as well as ISSs. For example, the sequence GTACATGT (supplementary table S5, motif #106) is the consensus silencer sequence (ISS motif) (p = 3.99e-04), while the sequence AGTTATCT (motif #5) is an enhancer (ISE) (p = 3.43e-04) (Figure 2A).

De-Novo Motifs & enriched sequences were identified using the HOMER (Hyper-geometric Optimization of Motif EnRichment) tool. The target sequences were selected based on the higher values of the average and maximal expression features, and the background sequences were selected based on the lower values of the average and maximal expression features and on non-spliced intron information, or vice-versa. We have identified 120 enriched motifs divided into 19 clans, based on 19 different comparisons of intron sets. The motifs were then coupled with all the introns sequences using each motif PSSM (Position-Specific Scoring Matrix). Each intron received a matching score accordingly.

A total number of 187 enriched motifs were found divided into 19 Clans. 175 motifs were significant (p-value <= 0.05). 151 motifs passed FDR (pN <= 6.354e-03). Additional 31 motifs were merged due to similarity, leaving a total of 120 motifs (See clan info in supplementary tables S4 and S5).

***Motif finding using HOMER***

HOMER is a motif discovery and sequencing analysis tool, written in Perl and C++ and run on a UNIX based OS platforms. It was designed for finding 8-20bp motifs in large scale genomics data. It is based on a differential motif discovery algorithm, *i.e.* it takes two sets of sequences and tries to identify the ***regulatory elements*** that are specifically enriched in one set relative to the other. It uses ZOOPS scoring (Zero or One Occurrence Per Sequence) coupled with the hyper-geometric enrichment calculations (or binomial) to determine motif enrichment.

The basic steps executed to discover regulatory elements are Preprocessing (steps 1-4), Discovering Motifs *De-Novo* (steps 5-9), and Motif Analysis and Presentation (steps10-12):

1. Extraction of Sequences.
2. Background Sequences Selection.
3. GC Normalization - sequences in the target and background sets are binned based on their GC-content (5% intervals). Background sequences are weighted to resemble the same GC-content distribution observed in the target sequences.
4. Auto-Normalization – Often the target sequences have an imbalance in the sequence content other than GC%. This technique is applied to full sequences (*i.e.* ~200bp) and is used to remove (or partially remove) imbalances in short oligo sequences (*i.e.* AA) by assigning weights to background sequences to help minimize the error.
5. Parsing input sequences into an Oligo Table.
6. Oligo Table Auto-Normalization (optional and done in a similar way to step 4).
7. Global search for Enriched "oligos" and calculation of Motif Enrichment - If a "Motif" is going to be enriched then the oligos considered part of the motif should also be enriched. Motif enrichment is calculated using either the cumulative hyper-geometric or cumulative binomial distributions.
8. Matrix Optimization - The most enriched oligos from the global optimization step, are transformed into simple PSSM, and further optimized with a sensitive local optimization algorithm. This step is performed separately for each oligo, and creates the "motif probability matrix" as well as determines the optimal detection threshold to maximize the enrichment of the motif in the target vs. background sequence.
9. Mask and Repeat - After the first "promising oligo" is optimized into a motif, the sequences bound by the motif are removed from the analysis and the next promising oligo is optimized for the 2nd motif, and so on. This is repeated until the desired number of motifs is found (default: 25).
10. Load Motif Library.
11. Screen Each Motif Enrichment - Each sequence is scanned for instances of the motif and the final enrichment is calculated by considering how many target *vs.* background sequences are considered "bound".  ZOOPS (zero or one occurrence per sequence) counting is used and the hyper-geometric or binomial is used to calculate the significance.

***Motifs scoring***

For each motif a set of matching scores were calculated for all intron. Each motif was described as a matrix of probabilities (PSSM); let $P_{i,j}^{f}$ be$P_{\mathrm{ij}}$ the probability that the i*th* nucleotide (*i.e.* C, T, G, or A) appears in the *jth* ( $1\leq j\leq k$ ) position of the motif $f$ ( $\left| f \right|=k$ is the length of the motifs). Let $S_{i}$ denote the nt at position *i* of the sequence$S$. The matching score of a certain nt sequence $S$ (of length $\left| f \right|=k$ ) to the motif $f$ is: $\sum_{j} log(P_{i,j}^{f})$. The score of an intron $l$ with respect to a motif $f$ $(\mathrm{Score}\left( f,l) \right)$ is the maximal matching score over all the subsequences of the intron to the motif$f$. Let $l_{i}^{j}$ denote the sub-intronic sequence between nt *i* and nt *j*. The score of the intron is computed according to the following formula:

$$Intron Score\left( f,l \right)={max}_{1\leq j\leq\left| l \right|-\left| f \right|+1}\left\{ \sum_{i=j}^{i+\left| f \right|-1} log(P_{l_{j},i-j+1}^{f}) \right\}$$

***Calculation of the motifs distribution***

Motifs distribution analysis was done by generating a set of random motifs that preserve the original motifs properties, by internal motif permutation tests. The location and significance level of the random motifs were calculated.

***Calculation of the distance between motifs***

Motifs distance calculation was done by comparing the probability matrices using the following formulation, which manages the expectations of the calculations by scrambling the nucleotide identities as a control; freq_1_ and freq_2_ are the matrices for motif_1_ and motif_2_, respectively:

$$Similarity Score=\frac{1}{Motif Length}\sum_{i}^{Motif Length} -\frac{{(Observed}_{i}-{Expect}_{i})}{{Expect}_{i}}$$

$${Observed}_{i}=\sum_{j}^{A,C,G,T} -\left( {freq}_{1}^{ij}-{freq}_{2}^{ij} \right)^{2}$$

$${Expect}_{i}=\sum_{j}^{A,C,G,T} \sum_{k}^{A,C,G,T} -\frac{\left( {freq}_{1}^{ij}-{freq}_{2}^{ik} \right)^{2}}{4}$$

Neutral frequencies (0.25) are used in where the motif matrices do not overlap. The output score ranges from some lower bound (depending on the matrix frequencies) to 1, where 1 is complete similarity. Empirically significant motifs with similarity score higher than 0.6 were merged. A total number of 55 motifs were merged (see supplementary table S5).

Randomization scores

Maximal randomized score: -0.3850 Maximal score: -0.3850

Minimal randomized score: -21.4273 Minimal score: -17.2060

Average randomized score: -4.8675 Average score: -4.4870

***Motif mutagenesis***

The intron transformation cassettes from strains YDL064W and YGL076C were ligated into the pGEM-T Easy vector (Promega) and the enhancer motif TTTATGCT (motif #70 in supplementary table S5) was mutated in three bases to match silencer motif #82 TTTGTGTA, using restriction free cloning^6^. The mutated transformation cassette were then transformed into the library master strain as previously described, and positive clones were verified by PCR and sequencing. The mutated motif introns (mut-i), the original YiFP, and the YFP-wt strains were cultured and splicing efficiency of the intron strains was calculated (Figure 2C).

**D.**

**Features database construction and expression analysis**

RNA secondary structure and folding energy

RNA secondary structure predictions were done using *rnafold* (Vienna) function. The function predicts and displays the secondary structure, associated with the minimum free energy for the RNA sequence, using the thermodynamic nearest-neighbor approach.

Folding Energy indicates the strength of the folding:

Energy = 0 - no folding

Energy = a < 0 - week folding

Energy = b < a < 0 - stronger folding

The GC Content and Folding Energy information was calculated for various sliding window sizes. 3 values are been used: 30bp (*i.e*. window A), 40bp (*i.e*. window B) and 50bp (*i.e*. window C). 2D structured distance calculations were done using RNA secondary structure predictions and the *Dijkstra* minimum path algorithm.

GC content and folding energy analysis

GC Content profiles in both splice sites were calculated for the yeast endogenous introns. They were compared to the YiFP synthetic library profiles. Folding Energy profiles in both splice sites and branch point were calculated for the yeast endogenous introns. They were compared to the YiFP synthetic library profiles.

Features list assembly

We developed a total of 1271 features that were constructed based on the following clustered sets:

1. Ares DB based – Intron Length; Branch Point (BP) Length; 2D structured Length from 5 'SS to the BP; 2D structured Length from BP to the 3' SS.
2. Folding Energy based – Average Folding Energy (FE) for windows A, B and C; Maximal FE for windows A, B and C; K Mean FE for windows A, B and C; FE Aligned to the 5'SS (*i.e*. FE_LA) for windows A, B and C; FE Aligned to the BP (*i.e*. FE_BP) for windows A, B and C; FE Aligned to the 3'SS (*i.e*. FE_RA) for windows A, B and C.
3. GC content based – GC Content Aligned to the 5'SS (*i.e*. GC_LA) for windows A, B and C; GC Content Aligned to the 3'SS (*i.e*. GC_RA) for windows A, B and C.
4. *De-novo* Motifs based – Motif Clan Scoring PSSM 1-19.

All the features were than analysis using various Machine learning & statistical tools. A linear regressor was built, using a greedy algorithm, to produce predictors for the various YFP expression level measurements (Details can be found in supplementary tables S7 and S8).

T-Test

The t-test method with no equal means (default: two-tailed test) was used to compare between Spliced and Non-Spliced introns based on various clustered features. The results are presented hereafter.

Correlations

The various features were interconnected with the expression level DB containing the normalized YFP fluorescence results. Correlation (Spearman) was done between the various expression level measurements and Ares/Folding/GC/Motifs databases. The correlation was only done for the spliced introns, with an additional subdivision to Ribosomal / Non-Ribosomal Introns.

Building a linear regressor

We constructed six regressors, divided into two sets for each measurement feature. Each set has a separate regressor for *Spliced*, *Spliced* *Ribosomal* and *Spliced Non-Ribosomal* introns .All the Ares, Folding, GC, and Motif prediction features were put into a linear regressor to assemble an expression predictor. The predictors were calculated for *spliced* introns and for *spliced* *Ribosomal* / *Non-Ribosomal* introns. For each predictor a feature assembly list was calculated. Accumulation of features was done using greedy algorithm. In each feature assembly iteration k, spearman correlation was calculated. The adjusted correlation value was calculated according to the following formula:

$R_{adjust}^{2}\left( k \right)=R^{2}-(1-R^{2})\frac{k}{n-(k+1)}$

, where *n* is the number of measurement features, and R is the Spearman correlation in the k-th iteration. The results were matched up to the value of R and are presented supplementary table 8.

**Statistics & Significance Level**

Randomization was done to determine the significance of the results. The cross validation was conducted in two phases: feature correlation level and regressor level.

Feature correlation cross validation

For every highly significant feature, an empirical p-value was calculated. The feature values were permutation and the feature to expression correlation was recalculated for 100 times. In most cases the empirical p-value was smaller than 0.01. Likewise the upper bound p-value was 0.03. The detailed results are presented in supplementary table S8.

Regressor cross-validation

For every regressor, cross validation was done and an empirical p-value was calculated. Each predictor values were permutation for 150 times, for each expression feature (*Avg*_*expr* and *Max_expr*). The basic correlations were calculated, and then they were put into a linear regressor to assemble an expression predictor. The original predictor results were compared to the randomized ones, and an empirical p-value was calculated. The results are presented in supplementary table S8.

In addition, the data was divided into training and test sets. We used 2 types of sets: 80/20 and 50/50 for the training and test respectively. Using the training set, de-novo motifs were found and the regressor was assembled based on all the features as described above. The resulting features were used to predict the test set values (Figure 4D).

Finally, we used the original predicator features to determine the strains new locations expression results. The GC content and FE features were revised according to new location information. The predictions were than correlated with the measured results (Figure 4E).

References:

1. Heinz S, Benner C, Spann N, Bertolino E, Lin YC, Laslo P, Cheng JX, Murre C, Singh H, Glass CK. 2010. Simple combinations of lineage-determining transcription factors prime. *Mol Cell* **38**(4): 576-589.
